# Correlates of domestic violence experience among recently-married women residing in slums in Pune, India

**DOI:** 10.1371/journal.pone.0195152

**Published:** 2018-04-02

**Authors:** Ameeta S. Kalokhe, Sandhya R. Iyer, Ambika R. Kolhe, Sampada Dhayarkar, Anuradha Paranjape, Carlos del Rio, Rob Stephenson, Seema Sahay

**Affiliations:** 1 Emory University School of Medicine, Department of Medicine, Division of Infectious Diseases, Atlanta, Georgia, United States of America; 2 Emory University Rollins School of Public Health, Department of Global Health, Atlanta, Georgia, United States of America; 3 National AIDS Research Institute, Department of Social and Behavioral Research, Pune, India; 4 National AIDS Research Institute, Department of Epidemiology and Biostatistics, Pune, India; 5 Temple University Lewis Katz School of Medicine, Department of Medicine, General Internal Medicine, Philadelphia, Pennsylvania, United States of America; 6 University of Michigan, School of Nursing, Department of Health Behavior and Biological Sciences, Ann Arbor, Michigan, United States of America; University of Westminster, UNITED KINGDOM

## Abstract

The high risk of experiencing domestic violence (DV) among married women in India who reside in slum communities underscores the need for effective, evidence-based, and culturally-tailored primary prevention. To inform such DV primary prevention strategies for this population, we herein aimed to identify correlates of DV experience in early marriage. Utilizing a cross-sectional design, potential correlates of DV experience were explored among a geographically-clustered random sample of 100 recently-married women residing in slums in Pune, India. In multivariable regression, DV experience was associated with less educational attainment by the participant’s spouse (standardized β = -0.281, p = 0.004), less satisfaction of the spouse’s family with the *maanpaan* (wedding-related gifts provided by the bride’s family) they received at the time of marriage (standardized β = -0.298, p<0.001), poorer conflict negotiation skills (standardized β = -0.308, p<0.001), and greater acknowledgement of DV occurrence in family and friends (standardized β = 0.436, p<0.001). These correlates suggest strategies that could be incorporated into future DV primary prevention interventions for this vulnerable population (i.e. promoting completion of formal education of boys alongside girls, mitigating causes of familial dowry harassment, improving conflict negotiation skills, and challenging norms surrounding DV).

## Introduction

Intimate partner violence (IPV), defined as the physical, sexual, psychological abuse, and control perpetrated against an intimate partner, is highly prevalent globally. Approximately one-third (30%) of women reporting physical and/or sexual abuse by their partner during their lifetime.[1] Not only is IPV a violation of human rights that often results in physical injury;[2] women who experience IPV have higher odds of depression, anxiety and other mental health disorders,[3,4] sexually transmitted infections including HIV,[5,6] chronic pain disorders,[4] and gynecologic morbidity [7] among other chronic disease states.[8,9] Additionally, their children suffer from greater morbidity and mortality.[10–12] In India, although national estimates suggest decreasing frequency, one in three women still report having been abused by their spouses during their lifetime.[13] Further, this figure is likely an underestimate of the abuse women suffer post-marriage, as it did not survey violence perpetration by the mother-in-law or other members of the husband’s family[14,15], hereafter termed domestic violence (DV).

Women who reside in India’s slums are among those at greatest risk of DV, with lifetime estimates of 21–99%.[16–21] While the disparate figures between slum- and non-slum residing communities may be in part artefactual due to shame-induced underreporting in higher income communities, factors that drive increased DV perpetration and compel women to remain in abusive relationships are likely disproportionately greater in slum communities. Women in slum communities may be more likely to experience DV because their partners and families into which they marry suffer greater stress (i.e. related to inadequate finances, crowding, limited resources, and poor sanitation), discrimination, and subordination, reside in communities where normalization and acceptance of DV is greater, alcohol use is greater, have weakened support systems that do not allow them to develop and exercise positive coping mechanisms, and use DV as means of countering feelings of powerlessness.[22,23] Further, in Pune slum communities, at the time of marriage, many women transition from nuclear to joint families and newly enter the slums from surrounding rural areas; thus, the differences in upbringing within the couple may also influence marital expectations and prompt conflict. Further, women residing in slums may be more likely to stay in abusive relationships because of poorer knowledge of and access to support services,[20] increased economic dependence,[19] weaker support systems,[19] stronger perceptions of hopelessness and disempowerment,[20,24] and residence in environments where DV and other forms of violence occurs with frequency and acceptance.[22,24] The risk imposed by these factors is compounded by social sanctions that encourage women to weaken ties with (and thus, diminish the social support of) natal family members and their community post-marriage, that limit the time the couple spends together alone to develop their relationship both pre- and post-marriage, and external pressure on the couple (i.e. fertility, socio-economic competition, pleasing of extended members of the marital family).[25–29] Further, women’s financial empowerment through employment, a seemingly logical solution, has counterintuitively been shown to be associated with increased DV experience through challenging traditional gender roles and serving as a “threat” to male partners.[17]

Thus, DV prevention for women residing in slum communities requires a culturally-tailored, community-tailored approach that recognizes the structural factors of slum environments that shape DV risk. Further, given the high DV burden and limited and saturated support resources, focus in resource-limited settings should be on primary prevention. National evidence suggests that almost two-thirds (62%) of women who report DV, state the abuse had begun within the first two years of marriage,[30] underscoring the need for such prevention efforts to occur pre- or immediately post-marriage. To date, few studies have examined risk factors for DV experience among women residing in slum communities in India.[16–20] Those who have, identified the following risk factors: age, low educational attainment of self and spouse, young age of marriage, having a love marriage versus arranged marriage, additional dowry request from marital family, employment, changes in her own or her spouse’s employment status, residence in a joint family, renting versus owning one’s residence, fewer rooms in the household and shared bathrooms, accepting attitudes toward wife beating, shorter duration of marriage, and spousal alcohol consumption.[16–21] And although causal directionality could not be established, one cross-sectional study among slum-dwelling women found participation in social groups and vocational training to be associated with DV experience, perhaps because participation challenges social norms.[21] Of note, none of these studies specifically examined correlates of violence in early marriage (i.e. 3–15 months), critical for primary DV prevention.

As part of formative work that led to the development of an intervention for the primary prevention of DV for newly-wed couples residing in Pune slums, we aimed to explore correlates of DV experience among recently-married women.

## Methods

### Ethics statement

This study was approved by the ethics committee of the National AIDS Research Institute (Pune, India, NARI-EC/2013-28) and the institutional review board of Emory University (Atlanta, USA, IRB00069846). All participants provided written informed consent prior to enrollment.

### Study design

The study was conducted in Pune, the second largest city in the western state of Maharashtra, India. According to most recent census data, the female-to-male sex ratio is 0.948, the average literacy among women is 87% and men 92%, and 22% live in slums.[31] Through our field experience, we learned that many women in the slums we sampled were first-generation migrants from the surrounding villages.

The study employed a cross-sectional design, wherein semi-structured interviews were conducted in one study visit. Interviews were conducted one-on-one in private by trained female study staff in either Marathi, Hindi, or English, as per the choice of the participant and lasted on average 70 minutes. Interviews were conducted in the participant’s home or place of work (contingent on assurance of privacy) or a nearby *anganwadi* (government childcare centers) or *mitra mandal* (youth center). A debriefing session was held with the women immediately after the interview to allow her to elaborate on her responses, ask questions of the interviewers, and inquire about potential support services.

#### Participant recruitment and enrollment

To be eligible for the study, participants had to be: 1) a woman over 18 years of age, 2) recently married (i.e. for 3–15 months), 3) in a first marriage, 4) residing with her spouse in a slum in Pune or Pimpri-Chinchwad (the two adjacent municipalities of Pune city), India, 5) planning to reside in the area for the majority of the following year, and 6) having oral fluency in either English, Marathi, or Hindi. Recruitment utilized geographically-clustered random sampling to sample slum areas from Pune and Pimpri-Chinchwad wards and convenience sampling to sample 4–5 women from within each slum area. Pune consists of 21 geographic wards (15 governed by the Pune Municipal Corporation and 6 governed by the Pimpri-Chinchwad Municipal Corporation). One slum was randomly chosen from each of these wards. If there were insufficient women willing and able to participate in the planned slum community, additional participants were recruited from a slum in the adjacent ward. Within slum communities, women were sampled using convenience sampling. Newly-married women were most often identified in collaboration with community key persons who knew the families within the slums well (i.e. *anganwadi* workers and members of community-based organizations); this approach was supplemented with 1) door-to-door recruitment (during which families were asked whether they had a newly-married women in their household) and 2) snowball sampling (where family members in a particular household were asked if they knew of a newly-married women in their community who may be interested in participating).

### Data collection

Data was collected using a 182-item semi-structured questionnaire (S1 Appendix) administered one-on-one in private by a trained female study team member. The questionnaire was informed by prior studies examining determinants of DV experience in other settings, piloted in 5 individuals, and modified accordingly to better achieve the aims of the study. The primary outcome, past 3-month experience of DV perpetrated by either the participant’s spouse or a member of the spouse’s family, was measured using the Indian Family Violence and Control Scale (IFVCS).[32] The IFVCS is a culturally-tailored DV measure, composed of 63 items informed by qualitative interviews with lay community members and individuals working directly with DV survivors in Pune[15], that surveys control, psychological, physical, and sexual abuse, and was validated in married women residing in Pune. Potential correlates of DV experience (Table 1) were surveyed across 6 domains: 1) socio-demographics, 2) DV conceptualization and acceptance, 3) the marital relationship and marital family relationship, 4) sexual communication and behaviors and sexual and reproductive health, 5) spousal substance abuse and gambling, and 6) stress, resilience, and social support.

**Table 1:** Correlates of DV experience among recently-married women living in Pune slum communities (n = 100).

Potential correlate	No. (%)	Correlation with DV total	Retained in domain model
*Domain 1*: *Socio-demographics*			
Age, mean (SD), *years*^†^	21.12 (2.45)	-0.199*	
Age of spouse, mean (SD), *years*^†^	25.56 (2.82)	-0.248*	
Age gap (self—spouse), mean (SD), *years*^†^	4.40 (2.60)	-0.074	
Education		-0.444***	
≤ Primary (7^th^ standard)	15 (15)		
Secondary (8^th^-10t standard)	38 (38)		
≥ Higher secondary (≥11 standard)	47 (47)		
Additional training	46 (46)	-0.148	
Education of spouse		-0.476***	X
≤ Primary (7^th^ standard)	14 (14)		
Secondary (8^th^-10t standard)	39 (39)		
≥ Higher secondary (≥11 standard)	47 (47)		
Additional training by spouse	26 (26)	-0.175	
Employment	5 (5)	-0.131	
Employment of spouse	99 (99)	-0.142	
Monthly income		-0.128	
None	95 (95)		
Rs. 0<x≤ 8000	3 (3)		
Rs. 8000<x≤ 10,000	1 (1)		
> Rs. 10,000	1 (1)		
Monthly income of spouse		-0.347***	**X**
None	1 (1)		
Rs. 0 <x≤ 8000	14 (14)		
Rs. 8000 <x≤ 10,000	17 (17)		
> Rs. 10,000	53 (53)		
Unknown	15 (15)		
Family type pre-marriage: nuclear	70 (70)	0.139	
Family type post-marriage: nuclear	14 (14)	-0.058	
Household members, mean (SD)	5.89 (2.90)	0.073	
Caste, reserved	48 (48)	0.026	
Religious affiliation			
Hindu	77 (77)	0.144	
Buddhist	14 (14)	0.091	
Muslim	5 (5)	-0.163	
Christian	4 (4)	-0.140	
Ever pregnant	54 (54)	0.048	
Had livebirth(s)	4 (4)	0.145	
Had planned abortion(s)	3 (3)	-0.033	
Had unplanned abortion(s)	8 (8)	-0.115	
Currently pregnant	44 (44)	0.074	
*Domain 2*: *DV conceptualization and acceptance*			
Household decision-making: mainly wife^†^	0.19 (0.13)	-0.032	
Household decision-making: both^†^	0.59 (0.21)	-0.205*	
Situational acceptance of wife-beating^†^	0.23 (0.24)	0.280**	**X**
Situational acceptance of wife’s sexual refusal^†^	2.97 (0.83)	-0.146	
Liberal definition of items constituting DV^‡^	3.56 (0.28)	-0.084	
Acknowledgement of DV occurrence in a friend/relative^‡^	0.18 (0.23)	0.539***	**X**
*Domain 3*: *The marital relationship and marital family relationship*			
Marital duration, mean (SD), *months*^†^	8.75 (3.57)	0.018	
Marriage type: arranged	73 (73)	-0.146	
Marriage within caste	83 (83)	-0.037	
Marriage within family	55 (55)	0.085	
Total face-to-face time with partner alone pre-marriage		-0.143	
None	49 (49)		
< 1 month	27 (27)		
1–6 months	5 (5)		
> 6 months	19 (19)		
Total time in contact with partner pre-marriage		-0.300**	
None	14 (14)		
< 1 month	26 (26)		
1–6 months	32 (32)		
> 6 months	28 (28)		
Extent of acquaintance with partner pre-marriage		-0.355***	**X**
Not at all	19 (19)		
Very little	20 (20)		
Somewhat	35 (35)		
Great extent	26 (26)		
Time spent with partner alone each week post-marriage		-0.235*	
Never	8 (8)		
Weekends/holidays only	37 (37)		
At least 3–4 days/week	55 (55)		
Greatest time spent working towards dreams of			
Spouse	9 (9)	**0.152**	
Self	7 (7)	**-0.094**	
Both	68 (68)	-0.301**	
Don’t	15 (15)	0.338***	
Greatest time spent discussing things of interest to			**X**
Spouse	8 (8)	0.353***	
Self	15 (15)	**-0.035**	
Both	72 (72)	-0.255**	
Don’t discuss things	5 (5)	**0.145**	
Great time spent doing things of interest to			**X**
Spouse	17 (17)	0.356***	
Self	14 (14)	**-0.102**	
Both	65 (65)	-0.257**	
Don’t do things	4 (4)	**0.126**	
Extent of attainment of the “wife ideal”		-0.249*	
≤ Very little	6 (6)		
Somewhat	48 (48)		
Great extent	45 (45)		
Extent of spouse’s attainment of the “husband ideal”		-0.406***	
≤ Very little	10 (10)		
Somewhat	30 (30)		
Great extent	59 (59)		
Satisfaction with future spouse at time of marriage		-0.211*	
≤ Very little	9 (9)		
Somewhat	24 (24)		
Great extent	65 (65)		
Satisfaction of spouse with *maanpaan* at time of marriage		-0.429***	
≤ Very little	6 (6)		
Somewhat	7 (7)		
Great extent	67 (67)		
Satisfaction of spouse’s family with *maanpaan* at time of marriage		-0.482***	**X**
≤ Very little	10 (10)		
Somewhat	11 (11)		
Great extent	61 (61)		
Satisfaction with in-law’s treatment since marriage		-0.366***	
≤ Very little	15 (15)		
Somewhat	18 (18)		
Great extent	63 (63)		
Parent’s satisfaction with spouse as a son-in-law		**-0.176**^+^	
≤ Very little	9 (9)		
Somewhat	9 (9)		
Great extent	80 (80)		
Conflict negotiation skills (CTS2n) ^‡^	3.28 (0.58)	-0.421***	**X**
Extent of jealousy if spouse talks to women within family		0.283**	
Never	68 (68)		
Rarely	10 (10)		
Sometimes	13 (13)		
Often	7 (7)		
Extent of jealousy if spouse talks to women outside family		0.167	
Never	56 (56)		
Rarely	11 (11)		
Sometimes	18 (18)		
Often	13 (13)		
*Domain 4*: *Sexual communication and behaviors*, *and sexual and reproductive health*			
Confidence in knowledge about sexual intercourse		-0.080	
Not at all	9 (9)		
Very little	43 (43)		
Somewhat	37 (37)		
Great extent	10 (10)		
Capacity to communicate unwillingness to have sex with partner		-0.247*	
Not at all	11 (11)		
Very little	7 (7)		
Somewhat	24 (24)		
Great extent	57 (57)		
Capacity to communicate willingness to have sex with partner		-0.259**	**X**
Not at all	20 (20)		
Very little	11 (11)		
Somewhat	23 (23)		
Great extent	45 (45)		
Last sexual intercourse			
Persuaded partner or partner persuaded	14 (14)	0.163	
Mutually willing (baseline group)	76 (76)	—-	
Partner forced me	6 (6)	0.098	
Prior use of a contraceptive	16 (16)	-0.158	
Prior discussion of contraceptive use with partner	28 (28)	-0.109	
Engagement in sexual relations outside of spouse	0 (0)	-	
*Domain 5*: *Spouse’s recent substance abuse and gambling*			
Spouse’s prior 3-month alcohol use		0.420***	**X**
Never	69 (69)		
Rarely	13 (13)		
Sometimes	14 (14)		
Often	3 (3)		
Spouse’s prior 3-month drug use	2 (2)	-0.028	
Spouse’s prior 3-month betting/gambling	1 (1)	0.333***	
*Domain 6*: *Stress*, *resilience*, *and social support*			
Stress due to financial trouble	33 (33)	0.110	
Stress due to non-continuous employment	21 (21)	0.280**	
Average number of scenarios causing stress^†^	0.22 (0.19)	0.457***	**X**
Perceived stress in past 3 months		0.416***	
Never	24 (24)		
Rarely	27 (27)		
Sometimes	36 (36)		
Often	12 (12)		
Resilience^‡^	3.25 (0.52)	-0.164	
Greatest support person if stressed: spouse	51 (51)	-0.192	
Supported by spouse if in conflict with family		-0.339***	**X**
≤ Rarely	17 (17)		
Sometimes	24 (24)		
Often	57 (57)		
Greatest support person(s) if marital conflict			
Parents	28 (28)	0.048	
Parents-in-law	44 (44)	-0.232*	
Other	24 (24)	0.149	
Perceived support from family if marital conflict		-0.334***	
≤ Very little	6 (6)		
Somewhat	15 (15)		
Great extent	77 (77)		

Column 1 describes the potential correlates that were assessed, Column 2 the distributions of the correlates, Column 3 the correlation for the respective bivariate analysis, and Column 4 indicates whether the correlate was ultimately retained in the respective domain model (which was run using variables significant at the bivariate level, choosing between highly collinear variables within the domain). Significant correlations are noted as follows

^+^p<0.10

*p≤0.05

**p≤0.01

***p≤0.001. Where test statistics are not followed by p-values, the correlations were not deemed significant. Variables designated with a † were analyzed as continuous variables, those designated with a “‡” were analyzed as available case means, and the remaining variables were categorical.

Where available, validated instruments were used to assess the potential correlates, with cultural adaptation as necessary. For example, the IFVCS was used to assess how participants conceptualized DV by asking to what extent they considered each of the 63 items to constitute violence. The 63 IFVCS items were similarly used to assess whether the participants were aware of DV occurring among their married female friends or relatives. To assess attitudes toward gendered household decision-making, we used the NFHS-3 decision-making module combined with 4 additional items examining decision-making regarding the healthcare of the wife, whether to have children, and whether and which contraception to use.[33] To assess attitudes toward acceptance of wife beating, the NFHS-3 Attitudes Toward Wife Beating questions[34] were used with the addition of three items assessing whether the participant felt beating was acceptable if a woman broke something expensive, was unable to conceive a child, or unable to have male child. The NFHS-3 Attitudes Toward Refusing Sexual Intercourse with Husband questions [34] were used to explore attitudes about woman’s autonomy related to sexual decision-making. The Negotiation Subscale of the Conflict Tactics Scale-2[35] was used to measure husband-wife conflict resolution skills and the Connor-Davidson Resilience Scale 10 (CD-RISC 10) was used to measure resilience.[36]

### Participant and study team safety

The study protocol was developed using the guidance of the WHO ethical and safety recommendations for research on violence against women.[37] Several measures were taken to protect the safety of the participants: 1) only female study staff recruited and interviewed the participants, 2) while the study was introduced as a “women’s health study” during community recruitment as per the WHO recommendations [37], participants were notified in private about the true study intent when they provided informed consent, 3) although a copy of the consent form was offered to all participants, they were encouraged to not keep it if they felt having the consent in their possession could jeopardize their safety, 4) the staff were responsible for ensuring the safety and privacy of the venue throughout the length of the interview and were trained in means of doing so (i.e. skipping the participant or discontinuing the interview if privacy could not be guaranteed, switching to general health questions if someone were to interrupt until privacy could again be ensured, having the second study staff member “keep watch” and “entertain” individuals who approached the home during the interview), 5) all participants were informed that they could skip any question or withdraw at any time if they did not feel comfortable, 6) all participants regardless of DV disclosure received a diary of support services and were offered facilitated referral to the services if needed, and 7) study data was de-identified and stored separately from informed consents (both in locked filing cabinets in a locked office).

Several measures were also undertaken to protect the safety of the staff: 1) the nearby police station was notified of the team’s presence in the field, 2) the team notified the research office when they entered and left the field, 3) staff always worked in pairs, 4) staff were trained to terminate the interview and leave the field if they ever felt their safety was in jeopardy, and 5) weekly debriefing meetings were held with the staff to discuss their field experiences and assess the impact on their personal mental health and need for support services or a break from the field.

### Statistical analysis

Data was entered into Microsoft Access, cleaned in Microsoft Excel, and transferred into SPSS for further analysis. Table 1 footnotes detail how each variable was operationalized. Measures of DV experience, definition, and experience by family and friends as well as resilience and conflict negotiation skills were calculated as available case means across all items of the respective scales to avoid most missing data scenarios. For the primary outcome variable of personal DV experience, as well the predictor variable ‘DV experience among family and friends,’ this represents a proportion because all items were binary. For DV definition, this represents an average that can be referred back to the ordinal scale for comparisons of severity (mild, moderate, etc). Related items among the predictors (such as the stress scale etc.) were combined in a similar fashion. Distributions of each of the potential DV predictors and outcome variable (past 3-month DV experience) were first assessed to justify the use of linear regression for DV experience and identify potential outliers (overly influential points) among predictors. Next significant correlates of DV experience were identified through bivariate analysis. All significant variables at the bivariate stage (p<0.05) were then examined within each domain for collinearity by examining the correlation matrix and looking for redundancy among predictors. If any two (or more) variables were considered collinear, variable selection was made based on the higher of the two correlations with the outcome. The next stage of data reduction was to allow the remaining significant predictors of DV experience to compete, *within each domain*, using multivariable regression models. The purpose of this was to find the most significant and *independent* predictors of DV experience within each domain before the domains were combined to complete a final model. This assures that only the most robust predictors are considered. Lastly, all variables significant in the domain-level models were included in a composite multivariable model with the exception of spousal drug use as it was reported with extremely low frequency. Backward elimination was then used to eliminate non-significant variables, resulting in the final DV experience prediction model. Control variables “age” and “education” were included in the final model for comparability with other studies.

## Results

### Study participants

We enrolled a random geographically-clustered sample of 100 recently-married women from slums in Pune (Table 1). One hundred and sixty-eight (168) women were approached to obtain the final sample of 100; among the 68 women who did not give consent to participate, 15 women were not permitted to participate by their mother-in-law, spouse, or other family member and 53 were not interested or lacked time availability. The average age was 21.12 years (range 18–30, σ = 2.45), greater than half had not completed education beyond the 10^th^ standard (53% or 53/100), the vast majority were unemployed (95% or 95/100) with no monthly income (95% or 95/100). Most lived in nuclear families pre-marriage (70% or 70/100), but lived in joint families post-marriage (86% or 86/100), with an average of 5.89 (σ = 2.90) household members. The majority reported Hinduism as their religious affiliation (77% or 77/100) and half self-reported that they belonged to the reserved category of caste (48% or 48/100). Since marriage (i.e. ≤18 months) over half (54% or 54/100) had been pregnant and 44% (44/100) were pregnant at the time of interview.

In contrast, the average age of the participants’ spouse was 25.56 years (range 20–33, σ = 2.82). And while the spouse’s education level was similar to that of the participant’s (53% or 53/100 not completing education beyond 10^th^ standard), almost all (99% or 99/100) were employed with half (53% or 53/100) earning greater than Rs. 10,000 (156 US Dollars) per month.

The average IFVCS scores (proportion of total DV types surveyed) for past 3-month experience of control, psychological, physical, and sexual DV were 0.51 (σ = 0.29), 0.09 (σ = 0.15), 0.03 (σ = 0.09), and 0.02 (σ = 0.08) respectively.

### Correlates of DV experience

In bivariate analysis (Table 1, Column 3), among all demographic factors more DV experience was associated with younger age of the participant (p≤0.05) and spouse (p≤0.05), less education of the participant (p≤0.001) and spouse, (p≤0.001), and lower monthly income of spouse (p≤0.001). In the DV conceptualization and acceptance domain, DV experience was associated with greater reporting of situations in which it was acceptable for a husband to beat his wife (p≤0.01) greater reporting of DV occurrence in a friend or relative (≤0.001) and less reporting of mutual decision-making as a couple (p≤0.05). Among the personal and family relationship variables, DV experience was associated with less contact and acquaintance before marriage (p≤0.01 and p≤0.001, respectively), spending less time together post-marriage (p≤0.05), not allotting time to work towards mutual dreams and goals (p<0.001), prioritizing time to discuss interests of the spouse (p< 0.001), and doing things solely of interest to the spouse (p≤0.001). DV experience was also associated with the participant reporting lower attainment of what society expected of her as a wife (p≤0.05) and her spouse as a husband (p≤0.001), being jealous of her spouse if he spoke with other women within the family (p≤0.01), and having poorer conflict negotiation skills (p≤0.001). Further, women who reported greater DV experience were less likely to report they were satisfied with their spouse at the time of marriage (p≤0.05), less likely to report that their spouse and his family were satisfied with the *maanpaan* they received at the time of marriage (p≤0.001 and p≤0.001, respectively), and less likely to report that they were satisfied by the way their in-laws treated them since marriage (p≤0.001).

In the sexual communication and sexual health domain, DV experience was associated with less capacity to communicate to one’s spouse unwillingness and willingness to have sex (p≤0.05, and p≤0.01, respectively). Among the variables exploring the spouse’s recent substance abuse and gambling, DV experience was associated with the participant reporting heavier alcohol use and gambling by her spouse in the prior 3 months (p≤0.001 and p≤0.001, respectively). Lastly, in the stress, resilience and social support domain, DV experience was associated with reporting of stress due to non-continuous employment (p≤0.01), identifying with more scenarios that cause stress (p≤0.001), and perceiving greater stress in the prior 3-months (p≤0.001), less support by her spouse if in conflict with his family (p≤0.001) and less support from her family if marital conflict were to occur (p≤0.001).

### Building the DV experience model

A number of variables were dropped from consideration due to collinearity. In choosing between variables, those with greater correlation with the outcome were used. For example, age and education of the respondent were both associated with age and education of the spouse; therefore, the spousal variables were used. Similarly, the amount of premarital contact was associated with how well the participant felt she knew her partner at marriage; the latter was used.

For items that indicated prioritizing couple time (i.e. to work toward goals, engage in discussions and activities), specifying prioritization of time to engage in activities of mutual interest was negatively correlated with prioritizing activities of sole interest to the spouse and with not dedicating time. Thus, for discussions and activities, the indicator variable ‘prioritization of interests of the spouse’ was used. For goals, ‘not prioritizing’ was used for further model selection. Capacity to communicate willingness and unwillingness to have sex were also highly correlated, so capacity to communicate willingness to have sex was used. Finally, in the stress domain the overall stress score was associated with prior 3 month stress, so the overall variable was used for further modeling.

Domain level models were next run using backward selection (p<0.05) to determine the significant and *independent* predictors in each domain. A multivariable model for Domain 4 was not run because after accounting for collinearity, only one variable, capacity to communicate willingness to have sex, was a candidate. Prior 3-month spousal betting and gambling was excluded from the final model because of the low frequency of affirmative response (1% or 1/100). The final model is shown in Table 2. In the final model, DV experience was significantly associated with less educational attainment by the participant’s spouse, less satisfaction of the spouse’s family with the *maanpaan* they received at the time of marriage, poorer conflict negotiation skills, and greater acknowledgement of DV occurrence in family and friends.

**Table 2:** Multivariable analysis exploring correlates of DV experience among newly-married women living in Pune slum communities (n = 100).

		Correlation with past 3-month experience of DV					
		Full combined model		Reduced combined model		Final model	
Domain	Predictor	Stand. β	p-value	Stand. Β	p-value	Stand. β	p-value
Control vars	Age					-0.095	0.224
	Education					0.120	0.243
Socio-demographics	Education of spouse	-0.176	0.054	-0.257	0.003	-0.281	0.004
	Monthly income of spouse	-0.118	0.150				
DV conceptualization and acceptance	Situational acceptance of wife-beating	0.002	0.986				
	Acknowledgement of DV occurrence in a friend/relative	0.331	<0.001	0.453	<0.001	0.436	<0.001
Marital relationship and marital family relationship	Extent of acquaintance with partner pre-marriage	-0.067	0.442				
	Prioritization of time spent discussing things of interest to spouse	0.077	0.349				
	Prioritization of time spent doing things of interest to spouse	0.113	0.158				
	Satisfaction of spouse’s family with *maanpaan* at time of marriage	-0.157	0.072	-0.192	0.023	-0.298	<0.001
	Conflict negotiation skills (CTS2n)	-0.197	0.034	-0.315	<0.001	-0.308	<0.001
Sexual communication and behaviors, and sexual and reproductive health	Capacity to communicate willingness to have sex with partner	-0.137	0.099				
Spouse’s recent substance abuse and gambling	Spouse’s prior 3-month alcohol use	0.106	0.252				
Stress, resilience, and social support	Percent of total possible scenarios causing stress	0.094	0.285				
	Supported by spouse if in conflict with family	-0.103	0.230				

Stand = standardized; vars = variables.

## Discussion

This study is the first to report correlates of DV experience in *early* marriage among women residing in slums in India, an advance critical for informing primary DV prevention for this vulnerable population. We identified four key potential DV correlates: low educational attainment by spouse, low marital family satisfaction with *maanpaan*, poor conflict negotiation capacity, and reporting knowledge of DV experience by close acquaintances. Our finding low educational attainment by the spouse to be a DV correlate is consistent with other studies in slum populations that have explored determinants of *lifetime* DV,[18,20] and suggests that long-term DV prevention efforts for low-income populations should consider furthering boys’ education alongside girls’ (the focus of present-day government programs).[38] Notably, instruction and training outside of formal education (i.e. vocational training, diploma courses) did not impact DV experience in our sample. Family dissatisfaction with dowry has also previously been associated with increased experience of abuse by women residing in slums.[21] Taken together with a recent study by Jeyaseelan *et al*[39] that linked dowry harassment to the husband’s low socio-economic status and his mother’s personal experience of dowry harassment, the evidence suggests that DV primary prevention interventions for such populations should incorporate components of financial empowerment for the couple to augment their income, and work with the family to challenge norms that propagate demand for dowry.

To the best of our knowledge, we are the first to identify poor conflict negotiation and acknowledgement of greater DV occurrence in friends and family as correlates of DV in this population. Identification of the prior is critical, because it is amenable to intervention: conflict negotiation skills can be taught and changed. Interestingly, acknowledgement of DV occurrence in close acquaintances was linked to increased personal DV experience even after controlling for attitudes toward acceptability of DV and the women’s definitions of behaviors constituting DV. This may be due to increased DV reporting due to less perceived DV-related stigma. An alternative possibility, supported by social learning theories, is that their male spouses are equally exposed to DV occurring in their families and friends, and that witnessing these experiences may contribute to their normalization of DV, therefore fueling their own perpetration of abuse[40].

Interestingly, some predictors of lifetime DV identified by other studies of slum-residing populations were not significantly correlated with DV experience in our population. This may be explained by the low variance in some variables (i.e. age, employment, family structure, marriage type, and marital duration). While low educational attainment was significantly associated with DV in the bivariate model, the association was likely not significant in the final model because it was included alongside spouse’s educational attainment, a significant correlate with which it was highly collinear. Other studies in similar populations have also found educational attainment of the male spouse to be as strong (if not stronger) a predictor of DV as education of the participant [18,20]. While prior studies have demonstrated accepting attitudes toward physical spousal abuse to be associated with increased DV experience in slum-based populations [16,19], they examined DV over a lifetime rather than in the initial months of marriage. The difference can be explained because normalization of DV is more likely to shape a woman’s likelihood of remaining in an abusive relationship (and thus incurring more DV over a lifetime) rather than her initial experience of DV as was measured in our study. While spousal alcohol abuse was associated with DV in bivariate analysis, our results were discrepant with other studies in similar populations [16,20] in that alcohol abuse was not significant in the multivariable analysis. The apparent association between spousal alcohol abuse and DV experience could be accounted for by controlling for the following confounders: educational attainment of spouse, familial dissatisfaction with dowry, the degree of spousal support the participant felt when in conflict with her spouse’s family, stress, prioritizing time to discuss interests of the spouse, and situational acceptance of physical abuse.

A major limitation of our study is the cross-sectional design which hindered our ability to examine the direction of association between the variables in the final model and DV experience. While spouse’s educational attainment and marital family satisfaction with *maanpaan* are easily justifiable as true predictors of DV because of their temporal relation to establishment of the marital relationship, the same is not the case for conflict negotiation skills and acknowledgement of DV in family and friends. For example, it is possible that experience of DV may result in the survivor losing her confidence and assertiveness in managing conflicts constructively[41]. Similarly, DV survivors may be more likely to report having close acquaintances who also have experienced DV to normalize their own experiences, or actually empathize with and befriend other individuals in similar situations. Another limitation of our study is a potential bias that results from selecting (and community peers identifying) women who were available and willing to participate (i.e. not restricted by their mothers-in-laws, spouses, or other family members as was the case for 15/168 women we approached). Perhaps, DV experience and the correlates of DV would have been different in women who lacked the autonomy to give consent to participate. Lastly, the study was originally designed to inform the development of a DV intervention and not specifically powered to detect differences in DV; however, as is evident from the results, the high levels of DV afforded us the opportunity for this unique analysis. In the future, results should be validated in a larger scale representative sample.

In conclusion, this study sheds light on potential predictors of DV experience in newly-married women residing in slums in India. In doing so, it suggests the need for DV primary prevention strategies for this vulnerable population to focus on promoting completion of formal education of both boys and girls (i.e. through scale-up of the Ministry of Human Resource Development national scholarship scheme for students from low-income families), reducing dowry harassment (i.e. through empowering the couple to seek alternate sources of income available through emerging government-sponsored vocational training and employment schemes and through challenging community norms regarding dowry), improving conflict negotiation capacity of young women (i.e. by integrating such training into the Kishori Shakti Yojana), and working with communities to help them expand their definition of behaviors constituting DV.

## Supporting information

S1 Appendix Survey instrument. (PDF)
