# Supplementary material for: Correlates of domestic violence experience among recently-married women residing in slums in Pune, India
Source: PLoS One. 2018 Apr 2;13(4):e0195152. doi: 10.1371/journal.pone.0195152 (PMC5880392; doi:10.1371/journal.pone.0195152)
Supplement: S1 Appendix — (PDF) [file pone.0195152.s001.pdf]

Healthy Relationships Survey (Women)

Participant ID: IPV-F-[ ][ ][ ] Date: ______________________
Interviewer's name :________________ Interviewer's signature ___________________
Start time _______________________
The first set of questions asks general questions about you, your spouse, and family. Please select the answer choice most fitting. As we proceed, let me know if you would like me to repeat a question at any point. Also let me know if you don't feel comfortable answering a particular question and we will move on. Are you ready to begin?
1. Age<br>• Years completed _________ ☐<br>• Estimated age (years completed)__________
2. Age of spouse<br>• Years completed _________<br>• Estimated age (years completed)__________<br>• Refuse to answer ______ 99
3. Highest level of education completed:<br>• Illiterate___________________________________1<br>• Functional literate ________________________ 2<br>• Primary education (Up to 7th standard) _______ 3<br>• Secondary education (8th – 10th standard) ____ 4<br>• Higher secondary education (11th-12th standard)__5<br>• Beyond graduate (12th pass) education_________ 6<br>• Refuse to answer___________________________99
4. Additional training completed (like technical degree, vocational training, diploma course)<br>• Yes______________________________________1<br>• No_______________________________________0<br>• Refuse to answer___________________________99
5. Highest level of education spouse completed:<br>• Illiterate___________________________________1<br>• Functional literate ________________________ 2<br>• Primary education (Up to 7th standard) _______ 3<br>• Secondary education (8th – 10th standard) ____ 4<br>• Higher secondary education (11th-12th standard)__5<br>• Beyond graduate (12th pass) education_________ 6<br>• Refuse to answer___________________________99

6. Additional training <u>spouse</u> completed (like technical degree, vocational training, diploma course)
   - Yes_______________________________________1
   - No_______________________________________0
   - Refuse to answer____________________________99

7. Participant employed?
   - Yes__________________________________,1 Specify________________
   - No_________ _________________________0
   - Refuse to answer _____________________99

8. <u>Spouse</u> employed?
   - Yes__________________________________,1 Specify_______________
   - No ______________________________0
   - Refuse to answer _____________________99

9. Monthly income of participant
   - NIL______________________0
   - Less than 2000___________1
   - Rs. 2000-4000____________2
   - Rs 4000-6000 ____________3
   - Rs. 6000-8000____________4
   - Rs. 8000-10000___________5
   - More than Rs 10000________6
   - I don't know______________97
   - Refuse to answer__________99

10. Monthly income of <u>spouse</u>
   - NIL______________________0
   - Less than 2000___________1
   - Rs. 2000-4000____________2
   - Rs 4000-6000 ____________3
   - Rs. 6000-8000____________4
   - Rs. 8000-10000___________5
   - More than Rs 10000________6
   - I don't know______________97
   - Refuse to answer__________99

11. Type of family (at present): *(read options aloud)*
   - Nuclear family ____1
   - Joint family ____2
   - Extended family____3
   - Extended/ joint family with separate kitchen _________________ 4
   - Live with friends____5
   - Refuse to answer____99

12. Number of members in household (at present)________

13. In what type of family did you spend <u>most</u> of your life before marriage? *(read options aloud)*
- Nuclear family ____1
- Joint family ____2
- Extended family____3
- Extended/ joint family with separate kitchen _________________ 4
- Lived with friends____5
- Orphanage____6
- Refuse to answer____99

14. Total number of pregnancies
- Pregnancies______________
- Refuse to answer__________99

(Should equal answer to #15. If pregnancies=0, then skip to #16)

15. Outcomes of pregnancies *[indicate number]*
- Live births_________________
- Planned abortions __________
- Unplanned abortions/still births______
- Currently pregnant (*'1' if yes, '0' if no*)______
- Refuse to answer____________99

16. Religion
- Hindu ________________________________ 1
- Muslim _______________________________ 2
- Christian______________________________ 3
- Bodh _________________________________ 4
- Jain__________________________________ 5
- Sikkh_________________________________6
- Atheist (no belief in existence of God) ____ 7
- Other (specify) _________________________8
- Refuse to answer _____________________ 99

The next questions ask about who in a marital relationship should have a greater say in various decisions. These questions are not necessarily about your relationship, but about marital relationships in general. We want to know that, in a couple, who you think should have the greatest say in each of the following decisions.

	Mainly the husband =1	Mainly the wife=2	Both equally= 3	Someone else=4	I don't know=97	Refuse to answer= 99
17. Her own health care	1	2	3	4	97	99
18. Major household purchases	1	2	3	4	97	99
19. Purchases for daily household needs	1	2	3	4	97	99
20. Visits to a wife's family or relatives	1	2	3	4	97	99
21. What to do with the money a wife earns from her work	1	2	3	4	97	99
22. Whether to have children	1	2	3	4	97	99
23. How many children to have	1	2	3	4	97	99
24. Whether to use contraception	1	2	3	4	97	99
25. Which contraception to use	1	2	3	4	97	99

Sometimes a husband is annoyed or angered by things his spouse does. In your opinion, is a husband justified in beating his spouse if in the following situations:

26. She goes out without telling him
- Yes________________1
- No_________________0
- Refuse to answer_____99

27. She neglects the house or children
- Yes________________1
- No_________________0
- Refuse to answer_____99

28. She argues with him
- Yes________________1
- No_________________0
- Refuse to answer_____99

29. She refuses to have sexual intercourse with him.
- Yes________________1
- No_________________0
- Refuse to answer_____99

30. She doesn't cook properly
- Yes________________1
- No_________________0
- Refuse to answer_____99

31. He suspects she is unfaithful.
- Yes________________1
- No_________________0
- Refuse to answer_____99

32. She shows disrespect for in-laws
- Yes________________1
- No_________________0
- Refuse to answer_____99

33. She breaks something expensive on purpose or by mistake
- Yes________________1
- No_________________0
- Refuse to answer_____99

34. The couple is unable to have a child
- Yes_______________1
- No________________0
- Refuse to answer____99

35. Why might it be justifiable for a husband to hit his wife if the couple is unable to have a child?
____
____
____

- Refuse to answer____99

36. The couple is unable to have a male child
- Yes_______________1
- No________________0
- Refuse to answer____99

37. Why might it be justifiable for a husband to hit his wife if the couple is unable to have a male child?

____
____
____

- Refuse to answer____99

So, now I’m going to give you different situations. In each situation a wife refused to have sex with her husband. Tell me how much you agree with her action.

	Not at all =1	Very little =2	Some what =3	To a great extent =4	Refuse to answer=99
38. She knew her husband has a sexually transmitted disease.	1	2	3	4	99
39. She knew her husband has sex with other women.	1	2	3	4	99
40. She was tired or not in the mood.	1	2	3	4	99

The next set of questions are a little bit tough and require concentration, but your answers are very important for us to understand the definition of violence. After this section, the survey will become light-hearted. Ready?

No matter how good a relationship we have with our family, sometimes there are disagreements or problems. The following questions ask about various freedoms a wife may experience during her married life. You will be given particular situations of curbing a wife's freedom. For each situation, I will ask you 3 questions:

1) To what extent should it be considered violence if a husband or his family does not allow his wife to engage in the freedom?
2) In your relatives or friends, are you aware of this restriction on a married woman?
3) In the past 3 months of your married life, how often have you been able to exercise the freedom without your husband or his family bothering you?

<table>
<tr><th>Situation</th><th colspan="5">To what extent should it be considered violence if a husband or his family does not allow his wife to [insert situation]?</th><th colspan="3">In your relatives or friends, are you aware of this restriction on a married woman?</th><th colspan="5">Thinking about the past 3 months of your married life how often have you been able to [insert scenario] without your husband or his family bothering you?</th></tr>
<tr><td></td><td colspan="8">Control over the following things:</td><td colspan="5">Freely able to do the following things:</td></tr>
<tr><td></td><td>Not violence =1</td><td>Mild violence =2</td><td>Moderate violence= 3</td><td>Severe violence =4</td><td>I don't know =97
N/A=98
Refuse to answer =99</td><td>Yes =1</td><td>No =0</td><td>I don't know =97
N/A=98
Refuse to answer =99</td><td>Never= 1</td><td>Rarely =2</td><td>Sometimes =3</td><td>Often =4</td><td>I don't know =97
N/A=98
Refuse to answer=9 9</td></tr>
<tr><td rowspan="2">41. Rest and relax when she wants to</td><td rowspan="2">1</td><td rowspan="2">2</td><td rowspan="2">3</td><td rowspan="2">4</td><td rowspan="2">DK97
NA98
R99</td><td rowspan="2">1</td><td rowspan="2">0</td><td rowspan="2">DK97
NA98
R99</td><td colspan="4">In the past 3 months, how often have you been able to rest and relax without your husband or his family bothering you?</td><td rowspan="2">DK97
NA98
R99</td></tr>
<tr><td>1</td><td>2</td><td>3</td><td>4</td></tr>
<tr><td rowspan="2">42. Spend her own or her self-earned money on her natal family.</td><td rowspan="2">1</td><td rowspan="2">2</td><td rowspan="2">3</td><td rowspan="2">4</td><td rowspan="2">DK97
NA98
R99</td><td rowspan="2">1</td><td rowspan="2">0</td><td rowspan="2">DK97
NA98
R99</td><td colspan="4">In the past 3 months, how often have you been able to spend your own or your self-earned money on your natal family without your husband or his family bothering you?</td><td rowspan="2">DK97
NA98
R99</td></tr>
<tr><td>1</td><td>2</td><td>3</td><td>4</td></tr>
</table>

	Not violence =1	Mild violence =2	Moderate violence= 3	Severe violence =4	DK97 NA98 R99	Yes =1	No =0	DK97 NA98 R99	Never= 1	Rarely =2	Sometimes =3	Often =4	DK97 NA98 R99
43. Spend her own or her self-earned money on **her children**.	1	2	3	4	DK97 NA98 R99	1	0	DK97 NA98 R99	In the <u>past 3 months</u>, how often have <u>you</u> been able to spend your own or your self-earned money on **your children** without your husband or his family bothering you? 1	2	3	4	DK97 NA98 R99
44. Spend her own or her self-earned money on **her friends**.	1	2	3	4	DK97 NA98 R99	1	0	DK97 NA98 R99	In the <u>past 3 months</u>, how often have <u>you</u> been able to spend your own or your self-earned money on **your friends** without your husband or his family bothering you?? 1	2	3	4	DK97 NA98 R99
45. Spend her own or her self-earned money on **her personal things**.	1	2	3	4	DK97 NA98 R99	1	0	DK97 NA98 R99	In the <u>past 3 months</u>, how often have <u>you</u> been able to spend your own or your self-earned money on **your personal things** without your husband or his family bothering you? 1	2	3	4	DK97 NA98 R99
46. Take up a new job or remain in her current job if she wants to.	1	2	3	4	DK97 NA98 R99	1	0	DK97 NA98 R99	In the <u>past 3 months</u>, how often have <u>you</u> been able to take up a new job or remain in your current job if you want to without your husband or his family bothering you? 1	2	3	4	DK97 NA98 R99

	Not violence =1	Mild violence =2	Moderate violence= 3	Severe violence =4	DK97 NA98 R99	Yes =1	No =0	DK97 NA98 R99	Never= 1	Rarely =2	Sometimes =3	Often =4	DK97 NA98 R99
47. Go out of the house.	1	2	3	4	DK97 NA98 R99	1	0	DK97 NA98 R99	In the past 3 months, how often have you been able to go out of the house without your husband or his family bothering you?				DK97 NA98 R99
									1	2	3	4	
48. Visit her natal family, friends, coworkers, relatives or other acquaintances.	1	2	3	4	DK97 NA98 R99	1	0	DK97 NA98 R99	In the past 3 months, how often have you been able to visit your natal family, friends, coworkers, relatives, or other acquaintances without your husband or his family bothering you?				DK97 NA98 R99
									1	2	3	4	
49. Talk freely on the phone, send SMS (text) messages, or use social media (like Facebook or WhatsApp).	1	2	3	4	DK97 NA98 R99	1	0	DK97 NA98 R99	In the past 3 months, how often have you been able to talk freely on the phone, send SMS (text) messages, or use social media (like Facebook or Whatsapp) without your husband or his family bothering you?				DK97 NA98 R99
									1	2	3	4	
50. Seek medical care for herself.	1	2	3	4	DK97 NA98 R99	1	0	DK97 NA98 R99	In the past 3 months, how often have you been able to seek medical care for yourself without your husband or his family bothering you?				DK97 NA98 R99
									1	2	3	4	

<table>
<tr><th></th><th>Not violence =1</th><th>Mild violence =2</th><th>Moderate violence= 3</th><th>Severe violence =4</th><th>DK97
NA98
R99</th><th>Yes =1</th><th>No =0</th><th>DK97
NA98
R99</th><th>Never= 1</th><th>Rarely =2</th><th>Sometimes =3</th><th>Often =4</th><th>DK97
NA98
R99</th></tr>
<tr><td rowspan="2">51. Make her own decisions about family-planning such as getting pregnant, using contraception, spacing between children, and permanent sterilization.</td><td rowspan="2">1</td><td rowspan="2">2</td><td rowspan="2">3</td><td rowspan="2">4</td><td rowspan="2">DK97
NA98
R99</td><td rowspan="2">1</td><td rowspan="2">0</td><td rowspan="2">DK97
NA98
R99</td><td colspan="4">In the <u>past 3 months</u>, how often have <u>you</u> been able to make your own decisions about family-planning such as getting pregnant, using contraception, spacing between children, and permanent sterilization without your husband or his family bothering you?</td><td rowspan="2">DK97
NA98
R99</td></tr>
<tr><td>1</td><td>2</td><td>3</td><td>4</td></tr>
<tr><td rowspan="2">52. Wear any type of dress and have any style that she wants besides sari.</td><td rowspan="2">1</td><td rowspan="2">2</td><td rowspan="2">3</td><td rowspan="2">4</td><td rowspan="2">DK97
NA98
R99</td><td rowspan="2">1</td><td rowspan="2">0</td><td rowspan="2">DK97
NA98
R99</td><td colspan="4">In the <u>past 3 months</u>, how often have <u>you</u> been able to wear any type of dress and have any style that you want besides sari without your husband or his family bothering you?</td><td rowspan="2">DK97
NA98
R99</td></tr>
<tr><td>1</td><td>2</td><td>3</td><td>4</td></tr>
<tr><td rowspan="2">53. Freely invite her natal family members and friends to visit her in her matrimonial home.</td><td rowspan="2">1</td><td rowspan="2">2</td><td rowspan="2">3</td><td rowspan="2">4</td><td rowspan="2">DK97
NA98
R99</td><td rowspan="2">1</td><td rowspan="2">0</td><td rowspan="2">DK97
NA98
R99</td><td colspan="4">In the <u>past 3 months</u>, how often have <u>you</u> been able to freely invite your natal family members and friends to visit your matrimonial home without your husband or his family bothering you?</td><td rowspan="2">DK97
NA98
R99</td></tr>
<tr><td>1</td><td>2</td><td>3</td><td>4</td></tr>
</table>

	Not violence =1	Mild violence =2	Moderate violence= 3	Severe violence =4	DK97<br>NA98<br>R99	Yes =1	No =0	DK97<br>NA98<br>R99	Never =1	Rarely =2	Sometimes =3	Often =4	DK97<br>NA98<br>R99
									In the past 3 months, how often have you been able to have sex how and when you want to without your husband or his family bothering you?				
54. Have sex how and when she wants to.	1	2	3	4	DK97<br>NA98<br>R99	1	0	DK97<br>NA98<br>R99	1	2	3	4	DK97<br>NA98<br>R99

The following questions again ask about the relationship between a husband and his wife or the husband's family and his wife. For each scenario we want to know:

1. To what extent should it be considered violence if a husband or a member of his family does one of the following to his wife?
2. In your relatives or friends are you aware of this happening to a married woman?
3. In the past 3 months of your married life has your husband or a member of his family done the following to you?

Situation	To what extent should it be considered violence if a husband or his family does one of the following to his wife [insert scenario]?					In your relatives or friends are you aware of this happening to a married woman?			In the past 3 months of your married life has your husband or his family done this to you?		
	Not violence=1	Mild violence=2	Moderate violence=3	Severe violence=4	I don't know =97<br>N/A=98<br>Refuse to answer =99	Yes=1	No=0	I don't know =97<br>N/A=98<br>Refuse to answer =99	Yes=1	No=0	I don't know =97<br>N/A=98<br>Refuse to answer =99
55. Screamed at her when she was alone.	1	2	3	4	DK97<br>NA98<br>R99	1	0	DK97<br>NA98<br>R99	1	0	DK97<br>NA98<br>R99

	Not violence=1	Mild violence=2	Moderate violence=3	Severe violence=4	DK97 NA98 R99	Yes=1	No=0	DK97 NA98 R99	Yes=1	No=0	DK97 NA98 R99
56. Excessively criticized her for her work at home	1	2	3	4	DK97 NA98 R99	1	0	DK97 NA98 R99	1	0	DK97 NA98 R99
57. Screamed at her or insulted her in front of others, in a public place, or on a social networking site.	1	2	3	4	DK97 NA98 R99	1	0	DK97 NA98 R99	1	0	DK97 NA98 R99
58. **Threatened** to send her out of the house.	1	2	3	4	DK97 NA98 R99	1	0	DK97 NA98 R99	1	0	DK97 NA98 R99
59. Forced her to leave the house.	1	2	3	4	DK97 NA98 R99	1	0	DK97 NA98 R99	1	0	DK97 NA98 R99
60. **Threatened** to send her to her natal home against her will.	1	2	3	4	DK97 NA98 R99	1	0	DK97 NA98 R99	1	0	DK97 NA98 R99
61. Sent her to her natal home against her will	1	2	3	4	DK97 NA98 R99	1	0	DK97 NA98 R99	1	0	DK97 NA98 R99

	Not violence=1	Mild violence=2	Moderate violence=3	Severe violence=4	DK97 NA98 R99	Yes=1	No=0	DK97 NA98 R99	Yes=1	No=0	DK97 NA98 R99
62. Harassed **her** for wedding-related gifts or money such as *maanpaan* or dowry.	1	2	3	4	DK97 NA98 R99	1	0	DK97 NA98 R99	1	0	DK97 NA98 R99
63. Harassed **her natal family** for wedding-related gifts or money such as *maanpaan* or dowry.	1	2	3	4	DK97 NA98 R99	1	0	DK97 NA98 R99	1	0	DK97 NA98 R99
64. Taunted her about her poor health.	1	2	3	4	DK97 NA98 R99	1	0	DK97 NA98 R99	1	0	DK97 NA98 R99
65. Threatened to hurt or hurt her **children** because the husband or his family were angry with her.	1	2	3	4	DK97 NA98 R99	1	0	DK97 NA98 R99	1	0	DK97 NA98 R99
66. Threatened to hurt or hurt a **member of her family** because the husband or his family were angry with her.	1	2	3	4	DK97 NA98 R99	1	0	DK97 NA98 R99	1	0	DK97 NA98 R99

	Not violence=1	Mild violence=2	Moderate violence=3	Severe violence=4	DK97 NA98 R99	Yes=1	No=0	DK97 NA98 R99	Yes=1	No=0	DK97 NA98 R99
67. Threatened to leave her and get remarried.	1	2	3	4	DK97 NA98 R99	1	0	DK97 NA98 R99	1	0	DK97 NA98 R99
68. Intentionally spread false rumors about her character and chastity.	1	2	3	4	DK97 NA98 R99	1	0	DK97 NA98 R99	1	0	DK97 NA98 R99
69. Intentionally ignored her or did not talk to her.	1	2	3	4	DK97 NA98 R99	1	0	DK97 NA98 R99	1	0	DK97 NA98 R99
70. Intentionally starved her or gave her stale food.	1	2	3	4	DK97 NA98 R99	1	0	DK97 NA98 R99	1	0	DK97 NA98 R99
71. Intentionally confined her in the house.	1	2	3	4	DK97 NA98 R99	1	0	DK97 NA98 R99	1	0	DK97 NA98 R99
72. Intentionally left her out of family functions or social events.	1	2	3	4	DK97 NA98 R99	1	0	DK97 NA98 R99	1	0	DK97 NA98 R99

	Not violence=1	Mild violence=2	Moderate violence=3	Severe violence=4	DK97 NA98 R99	Yes=1	No=0	DK97 NA98 R99	Yes=1	No=0	DK97 NA98 R99
73. Bothered her for having a girl child.	1	2	3	4	DK97 NA98 R99	1	0	DK97 NA98 R99	1	0	DK97 NA98 R99
74. Bothered her for being infertile.	1	2	3	4	DK97 NA98 R99	1	0	DK97 NA98 R99	1	0	DK97 NA98 R99
75. Forced her to become vegetarian or non-vegetarian.	1	2	3	4	DK97 NA98 R99	1	0	DK97 NA98 R99	1	0	DK97 NA98 R99
76. Forced her to fast (perform *upvas*) when she did not want to.	1	2	3	4	DK97 NA98 R99	1	0	DK97 NA98 R99	1	0	DK97 NA98 R99
77. Forced her to work excessively against her will.	1	2	3	4	DK97 NA98 R99	1	0	DK97 NA98 R99	1	0	DK97 NA98 R99
78. Slapped or scratched her.	1	2	3	4	DK97 NA98 R99	1	0	DK97 NA98 R99	1	0	DK97 NA98 R99

	Not violence=1	Mild violence=2	Moderate violence=3	Severe violence=4	DK97 NA98 R99	Yes=1	No=0	DK97 NA98 R99	Yes=1	No=0	DK97 NA98 R99
79. Kicked, punched, or beat her.	1	2	3	4	DK97 NA98 R99	1	0	DK97 NA98 R99	1	0	DK97 NA98 R99
80. Twisted her arm or pulled her hair.	1	2	3	4	DK97 NA98 R99	1	0	DK97 NA98 R99	1	0	DK97 NA98 R99
81. Pushed her, pulled her, dragged her, shook her, or held her down.	1	2	3	4	DK97 NA98 R99	1	0	DK97 NA98 R99	1	0	DK97 NA98 R99
82. Tried to strangle or suffocate her.	1	2	3	4	DK97 NA98 R99	1	0	DK97 NA98 R99	1	0	DK97 NA98 R99
83. Tried to hang her.	1	2	3	4	DK97 NA98 R99	1	0	DK97 NA98 R99	1	0	DK97 NA98 R99
84. Tried to poison her.	1	2	3	4	DK97 NA98 R99	1	0	DK97 NA98 R99	1	0	DK97 NA98 R99

	Not violence=1	Mild violence=2	Moderate violence=3	Severe violence=4	DK97 NA98 R99	Yes=1	No=0	DK97 NA98 R99	Yes=1	No=0	DK97 NA98 R99
85. Threw things in the house when he or his family were angry with her.	1	2	3	4	DK97 NA98 R99	1	0	DK97 NA98 R99	1	0	DK97 NA98 R99
86. Burnt her or threatened to burn her with a cigarette or bidi.	1	2	3	4	DK97 NA98 R99	1	0	DK97 NA98 R99	1	0	DK97 NA98 R99
87. **Threatened** to burn her using kerosene, chemicals, acid, or some other method.	1	2	3	4	DK97 NA98 R99	1	0	DK97 NA98 R99	1	0	DK97 NA98 R99
88. Burned her using kerosene, chemicals, acid, or some other method.	1	2	3	4	DK97 NA98 R99	1	0	DK97 NA98 R99	1	0	DK97 NA98 R99
89. **Threatened** her with a **sharp object** such as broken glass, a razor blade, axe, or knife.	1	2	3	4	DK97 NA98 R99	1	0	DK97 NA98 R99	1	0	DK97 NA98 R99
90. Attacked her with a **sharp object** such as broken glass, a razor blade, axe, or knife.	1	2	3	4	DK97 NA98 R99	1	0	DK97 NA98 R99	1	0	DK97 NA98 R99

	Not violence=1	Mild violence=2	Moderate violence=3	Severe violence=4	DK97 NA98 R99	Yes=1	No=0	DK97 NA98 R99	Yes=1	No=0	DK97 NA98 R99
91. **Threatened** her with a **blunt object** such as a belt, stone, broomstick, or rolling pin.	1	2	3	4	DK97 NA98 R99	1	0	DK97 NA98 R99	1	0	DK97 NA98 R99
92. Attacked her with a **blunt object** such as a belt, stone, broomstick, or rolling pin.	1	2	3	4	DK97 NA98 R99	1	0	DK97 NA98 R99	1	0	DK97 NA98 R99
93. Forced her to have sex against her will during her **menstrual cycle**.	1	2	3	4	DK97 NA98 R99	1	0	DK97 NA98 R99	1	0	DK97 NA98 R99
94. Forced her to have sex against her will with **someone else**.	1	2	3	4	DK97 NA98 R99	1	0	DK97 NA98 R99	1	0	DK97 NA98 R99
95. Purposely made her drunk or high on drugs to force her to have sex against her will.	1	2	3	4	DK97 NA98 R99	1	0	DK97 NA98 R99	1	0	DK97 NA98 R99
96. Forced her to have sex without a **condom** against her will.	1	2	3	4	DK97 NA98 R99	1	0	DK97 NA98 R99	1	0	DK97 NA98 R99

	Not violence=1	Mild violence=2	Moderate violence=3	Severe violence=4	DK97 NA98 R99	Yes=1	No=0	DK97 NA98 R99	Yes=1	No=0	DK97 NA98 R99
97. Forced her to replicate a sexual behavior from a pornographic film against her will.	1	2	3	4	DK97 NA98 R99	1	0	DK97 NA98 R99	1	0	DK97 NA98 R99
98. Forced her to engage in **vaginal** sexual intercourse against her will.	1	2	3	4	DK97 NA98 R99	1	0	DK97 NA98 R99	1	0	DK97 NA98 R99
99. Forced her to engage in **oral** sex against her will.	1	2	3	4	DK97 NA98 R99	1	0	DK97 NA98 R99	1	0	DK97 NA98 R99
100. Forced her to engage in **anal** sex against her will.	1	2	3	4	DK97 NA98 R99	1	0	DK97 NA98 R99	1	0	DK97 NA98 R99
101. Videotaped her and her husband having sex against her will	1	2	3	4	DK97 NA98 R99	1	0	DK97 NA98 R99	1	0	DK97 NA98 R99
102. Intentionall y performed forceful sex to hurt her.	1	2	3	4	DK97 NA98 R99	1	0	DK97 NA98 R99	1	0	DK97 NA98 R99

	Not violence=1	Mild violence=2	Moderate violence=3	Severe violence=4	DK97<br>NA98<br>R99	Yes=1	No=0	DK97<br>NA98<br>R99	Yes=1	No=0	DK97<br>NA98<br>R99
103. Threatened to sexually abuse someone that she cares about if she refused to have sex.	1	2	3	4	DK97<br>NA98<br>R99	1	0	DK97<br>NA98<br>R99	1	0	DK97<br>NA98<br>R99

The next few questions ask about your marriage.

104. Of the following which best describes your marriage? (read options aloud)

- Arranged ___________________ 1
- Love with acceptance by family___2
- Love without acceptance of family_3
- Refuse to answer_____99

105. Caste:

Marriage			
105a. Pl mention:<br><br>Within Caste________1<br><br>Intercaste__________2<br><br>I don't know________97<br><br>Refuse to answer____99<br><br>*If inter-caste, skip to 105c*	105b. Marriage within family relations?<br><br>Yes_____1<br><br>No_____0<br><br>Refuse to answer ____99	105c. Name of the caste through marriage<br>______________________<br>______________________<br><br>Refuse to answer______99	105d. Is the caste:<br><br>Reserved_________1<br><br>Non-reserved_____2<br><br>I don't know______97<br><br>Refuse to answer______99

106. Prior to marriage how much time did you spend alone with your partner (current spouse) face-to-face?

- Duration (___minutes / _____hours/_____ days / _____months / _____ years)
- Refuse to answer___99

107. Prior to marriage how long had you been in contact (for example, by phone, chatting, Whatsapp)?

- Never___________________________________1
- 0 <X ≤2 weeks____________________________2
- 2<X ≤4 weeks_____________________________3
- 1<X ≤3 months____________________________4
- 3<X ≤6 months____________________________5
- 6<X ≤9 months____________________________6
- 9<X ≤12 months___________________________7
- More than 12 months (specify)________________8
- Refuse to answer___________________________99

108. How well did you feel you knew your partner (current spouse) at the time of marriage? *(read options aloud)*

- Not at all____________ 1
- Very little____________2
- Somewhat __________ 3
- To a great extent _____ 4
- Refuse to answer _____99

109. What have you learned about your spouse that you didn't know at the time of marriage?

____

____

____

The next set of questions ask about how you spend your time with your spouse at present.

110. On average, which best describes how much time (outside of sleep) you and your spouse spend together alone?

- Never______________________________1
- Every day __________________________2 (specify hours per day) ________
- 3-4 days each week__________________ 3 (specify hours per day) ________
- Only on weekends and holidays_________4 (specify hours per day)_________
- Refuse to answer_____________________99

111. What are the major things you want to accomplish in your life?

- ____
  ____
- I don't know____97
- Refuse to answer_____________________________________99

112. What are the major things your spouse wants to accomplish in his life?

- ____
  ____
- I don't know____97
- Refuse to answer_____________________________________99

113. Thinking about the time you and your spouse spend together and the dreams and goals you just told me about, whose dreams do you prioritize working on?

- The dreams/goals of my spouse_______________________________1
- My dreams/goals ____2
- Both of our dreams/goals _____________________________________3
- We do not spend time working toward our dreams or goals____________4
- I don't know____97
- Not applicable (We do not dream)______________________________98
- Refuse to answer ____99

Of the following activities you and your spouse may do together, usually, to whose liking and interest are they?

	Of interest to my spouse=1	Of interest to me=2	Of interest to both of us=3	We do not really discuss/do this=4	Refuse to answer=99
114. Discuss things	1	2	3	4	99
115. Do things or activities	1	2	3	4	99

116. What are the recreational activities where only the two of you are involved? (*probe*)
____
____

We will now talk about the desired qualities of a husband and wife. I want you to take a minute and picture an "ideal wife" and "ideal husband."

117. Please tell me the 3 most important qualities (i.e. attributes or model behaviors) of an "ideal wife"

1.________________________

2.________________________

3.________________________

118. Please tell me the extent to which you feel you have attained these:

- Not at all_____________ 1
- Very little_____________2
- Somewhat ___________ 3
- To a great extent _____ 4
- Refuse to answer _____99

119. Please tell me the 3 most important qualities (i.e. attributes or model behaviors) of an "ideal husband"

1.________________________

2.________________________

3.________________________

120. Please tell me the extent to which you feel your spouse has attained these:

- Not at all____________ 1
- Very little____________2
- Somewhat __________ 3
- To a great extent _____ 4
- Refuse to answer _____99

The following questions ask you about your marriage and relationship with your family. We would like to know the level of satisfaction with the following aspects of your marriage.

	Not at all =1	Very little =2	Somewhat =3	To a great extent =4	I don't know =97	Not applicable =98	Refuse to answer =99
121. Your satisfaction with your future life partner (current spouse) at the time of marriage	1	2	3	4	97	98	99
122. Satisfaction of your spouse with wedding-related gifts or *maanpaan* provided at the time of marriage	1	2	3	4	97	98	99
123. Satisfaction of your spouse's family with wedding-related gifts or *maanpaan* provided at the time of marriage	1	2	3	4	97	98	99
124. Your satisfaction with how your in-laws have treated you since marriage	1	2	3	4	97	98	99
125. Your parents' satisfaction with your spouse as a son-in-law since marriage	1	2	3	4	97	98	99

The next set of questions asks about how you handled disagreements with your spouse in the past 3 months. Thinking about the past 3 months when you and your spouse had disagreements, please choose how often you did the following.							
	Never=1	Rarely=2	Sometimes=3	Often=4	I don’t know=97	Not applicable=98	Refuse to answer=99
126. I showed my spouse I cared even though we disagreed	1	2	3	4	97	98	99
127. I showed respect for my spouse’s feelings about an issue	1	2	3	4	97	98	99
128. I said I was sure we could work out a problem	1	2	3	4	97	98	99
129. I explained my side of a disagreement to my spouse	1	2	3	4	97	98	99
130. I suggested a compromise to a disagreement	1	2	3	4	97	98	99
131. I agreed to try a solution to a disagreement my spouse suggested	1	2	3	4	97	98	99
132. How often do you get upset if your spouse talks with other women within the family?	1	2	3	4	97	98	99
133. How often do you get upset if your spouse talks with other women outside the family?	1	2	3	4	97	98	99

The next few question ask about relationships, sex, and reproductive health.

134. What were the 3 most important sources of information for you about relationships between a husband and wife? *(do not read options aloud)*

- school teacher or school program_______1
- spouse______________________________2
- mother______________________________3
- father_______________________________4
- brother______________________________5
- sister________________________________6
- other family members_________________7
- friends______________________________8
- doctor, nurse, or counselor_____________9
- books/magazines____________________10
- films/videos__________________________11
- internet_____________________________12
- mobile clips__________________________13
- other (specify)______________________14
- I received no information on this topic____15
- Refuse to answer____________________99

135. What were the 3 most important sources of information for you on sexual and reproductive systems of men and women and sexual intercourse? *(do not read options aloud)*

- school teacher or school program_______1
- spouse______________________________2
- mother______________________________3
- father_______________________________4
- brother______________________________5
- sister________________________________6
- other family members_________________7
- friends______________________________8
- doctor, nurse or counselor_____________9
- books/magazines____________________10
- films/videos__________________________11
- internet_____________________________12
- mobile clips__________________________13
- other (specify)______________________14
- I received no information on this topic____15
- Refuse to answer____________________99

Please tell me the extent to which you agree with the following statements.

	Not at all=1	Very little=2	Somewhat =3	To a great extent=4	Refuse to answer =99
136. I feel confident about my knowledge of sexual intercourse.	1	2	3	4	99
137. I can let my spouse know when I do not want to have sex with him.	1	2	3	4	99
138. I can let my spouse know when I do want to have sex with him.	1	2	3	4	99

139. Think back to the last time you had sex with your spouse, which of the following statements is most fitting: *(read options aloud)*
- I persuaded him to have intercourse____________________1
- He persuaded me to have intercourse____________________2
- We were both equally willing__________________________3
- I had intercourse with him against his will________________4
- He had intercourse with me against my will_______________5
- We have not been able to consummate our marriage_______6
- Refuse to answer___________________________________99

140. Have you ever used any contraceptive?
- Yes___________________1
- No____________________0
- Refused to answer_______99

141. Did you ever discuss contraception with your spouse?
- Yes______________________1
- No_______________________0
- Refuse to answer___________99

142. Have you ever had sexual relations with someone outside of your spouse?
- Yes_____________________1
- No______________________0
- Refuse to answer__________99

The next section asks about <u>your spouse's</u> use of alcohol, drugs and gambling. I want you to tell me which option <u>best</u> describes how often <u>your spouse</u> has done the following in the <u>past 3 months</u>.						
	Never =1	Rarely =2	Sometimes=3	Often=4	I don't know =97	Refuse to answer =99
143. Alcohol or related fermented drinks (like *haat bhatti* or desi daru)	1	2	3	4	97	99
144. Drugs or mind-altering substances (like *ganja*, sniffing whitener, raw ink, Iodex, *bhang* or other)	1	2	3	4	97	99
145. Bet for money or gambled	1	2	3	4	97	99

We have now reached the final set of questions. The next section asks about stress in your life and how you deal with it. Are you ready?

First, I am going to read you a couple of scenarios or problems. Please tell me whether you have experienced stress due to them in the <u>past 3 months</u>.			
	Yes=1	No=0	Refuse to answer=99
146. Financial trouble or debt	1	0	99
147. A land dispute/court case	1	0	99
148. Not having continuous employment	1	0	99
149. Your poor health	1	0	99
150. Your spouse's poor health	1	0	99
151. Illness or death in your family	1	0	99
152. Problems with marital relations	1	0	99
153. Problems with familial relations	1	0	99
154. Difficulty with bearing children	1	0	99
155. Abortion/s	1	0	99
156. Mistreatment or abuse by your boss at the workplace	1	0	99
157. Discrimination or poor treatment because of caste, religion, or migrant status	1	0	99
158. Problems with access to water, electricity, gas, or toilet facility	1	0	99
159. Dislocation or eviction from your home	1	0	99
160. Crowded or non-hygienic living conditions	1	0	99
161. Neighborhood crime or problems with the safety of your family	1	0	99
162. Noise	1	0	99
163. Other____ ____________________________________	1	0	99

164. During the past 3 months how often have you felt stressed (tension)?

- Never_____________1
- Rarely_____________2
- Sometimes_________3
- Often______________4
- Refuse to answer ____99

Please indicate how much you agree with the following statements as they apply to you over the last month. If a particular situation has not occurred recently, answer according to how you think you would have felt.

165. I am able to adapt when change occurs

- Not true at all________________1
- Rarely true__________________2
- Sometimes true______________3
- Often true___________________4
- True nearly all the time_________5
- Refuse to answer ____________99

166. I can deal with whatever comes my way

- Not true at all________________1
- Rarely true__________________2
- Sometimes true______________3
- Often true___________________4
- True nearly all the time_________5
- Refuse to answer ____________99

167. I try to see the humorous side of things when I am faced with problems.

- Not true at all________________1
- Rarely true__________________2
- Sometimes true______________3
- Often true___________________4
- True nearly all the time_________5
- Refuse to answer ____________99

168. Having to cope with stress can make me stronger

- Not true at all________________1
- Rarely true__________________2
- Sometimes true______________3
- Often true___________________4
- True nearly all the time_________5
- Refuse to answer ____________99

169. I tend to bounce back after illness, injury or other hardships

- Not true at all________________1
- Rarely true__________________2
- Sometimes true______________3
- Often true___________________4
- True nearly all the time_________5
- Refuse to answer ____________99

170. I believe I can achieve my goals, even if there are obstacles

- Not true at all________________1
- Rarely true__________________2
- Sometimes true______________3
- Often true___________________4
- True nearly all the time_________5
- Refuse to answer ____________99

171. Under pressure, I can stay focused and think clearly.

- Not true at all________________1
- Rarely true__________________2
- Sometimes true______________3
- Often true___________________4
- True nearly all the time_________5
- Refuse to answer ____________99

172. I am not easily discouraged by failure

- Not true at all________________1
- Rarely true__________________2
- Sometimes true______________3
- Often true___________________4
- True nearly all the time_________5
- Refuse to answer ____________99

173. I think of myself as a strong person when dealing with life's challenges and difficulties.

- Not true at all________________1
- Rarely true__________________2
- Sometimes true______________3
- Often true___________________4
- True nearly all the time_________5
- Refuse to answer ____________99

174. I am able to handle unpleasant or painful feelings like sadness, fear, and anger.

- Not true at all________________1
- Rarely true__________________2
- Sometimes true______________3
- Often true___________________4
- True nearly all the time_________5
- Refuse to answer ____________99

175. People often experience events that cause stress and deal with them in different ways. Please tell me the two most common ways you deal with stress:

1. ____
____
2. ____
____

176. Who is the one person you feel most comfortable talking to about stress or trouble in your life:

- Friend_______________________1
- Mother_______________________2
- Father_______________________3
- Sibling_______________________4
- In-laws_______________________5
- Spouse______________________6
- Doctor, nurse, or counselor_______7
- Other________________________8 (specify)_________________________
- Nobody_____________________9
- Refuse to answer______________99

177. When there is conflict between you and your spouse's parents or you and other members of your spouse's family how often does your spouse support you?

- Never____________ 1
- Rarely_____________2
- Sometimes_________ 3
- Often________ _____ 4
- Not applicable________98
- Refuse to answer _____99

178. If there were a problem with your marriage (your husband-wife relationship), who would be the one biggest source of support for you?

- Friend________________________1
- Mother________________________2
- Father________________________3
- Sibling________________________4
- In-laws________________________5
- Other_________________________6 (specify)__________________________
- Nobody______________________7
- Refuse to answer______________99

179. If there were trouble in your marriage (your husband-wife relationship), how supported do you think you would feel by your parents and other members of your own family?

- Not at all____________ 1
- Very little____________2
- Somewhat __________ 3
- To a great extent _____ 4
- Refuse to answer _____99

Lastly, I want you to think a little bit more about your relationship with your spouse.

180. What are the main causes of disharmony in your marriage?

____
____
____

181. What are the main causes of happiness in your marriage?

____
____
____

182. If you could change one thing about your marriage today, what would it be?

____
____
____

Debriefing Notes:

____
____
____
____
____

- *Ask participant if they have any questions about the study and attempt to answer to the best of your ability.*
- *Clarify misconceptions regarding health knowledge.*
- *Offer to help refer the participants to the local community resources. Provide her with the phone diary and explain it.*

End time_________________

Phone diary:

- ☐ Participant accepted
- ☐ Participant declined

Coupons:

- ☐ Participant accepted
- ☐ Participant declined

QC completed by interviewer before leaving field: *(initials)* ____ ____ ____

QC completed by DEO:

- ☐ Date______/______/_____

QC completed by RS-1:

- ☐ Date______/______/_____

- All rights are reserved on the aforementioned RISC-10 Scale (questions 165-174). No part of this document may be reproduced or transmitted in any form, or by any means, electronic or mechanical, including photocopying, or by any information storage or retrieval system, without permission from Dr. Davidson at mail@cd-risc.com. Further information about the scale and terms of use can be found at www.cd-risc.com. Copyright ©2001, 2014 by Kathryn M. Connor, M.D., and Jonathan R.T. Davidson, M.D. This version of the scale was developed as a work made for hire by Laura Campbell-Siles, PhD., and Murray B. Stein, M.D.

Important definitions for the interviewer/reader:

- *A husband's family= his mother, his father, his brother, or his sister.*
- *Spouse's natal family= the family into which the spouse was born. That includes her mother, her father, her brother, or her sister.*
- *Sex=either vaginal, anal, or oral sex unless we specify a specific type of sex in the question.*
